# Reduction in Processing Time in Ca_3_Co_4_O_9+δ_ Ceramics through Nanoprecursors Produced by an Easily Scalable and Environmentally Friendly Process

**DOI:** 10.3390/nano10122533

**Published:** 2020-12-17

**Authors:** Hippolyte Amaveda, Maria A. Madre, Mario Mora, Miguel A. Torres, Andres Sotelo

**Affiliations:** Instituto de Nanociencia y Materiales de Aragón (CSIC-Universidad de Zaragoza), Mª de Luna, 3, 50018 Zaragoza, Spain; hippo@unizar.es (H.A.); amadre@unizar.es (M.A.M.); mmora@unizar.es (M.M.); matorres@unizar.es (M.A.T.)

**Keywords:** ceramics, oxides, electric properties, microstructure, power factor

## Abstract

Attrition milling is an easily scalable and environmentally friendly process used to produce Ca_3_Co_4_O_9+δ_ nanoprecursors in a relatively short time. Sintered materials produced through the classical solid-state method, involving ball milling, show much larger grain sizes and slightly lower density than those obtained in samples produced from attrition-milled precursors. On the other hand, electrical resistivity has been drastically decreased, accompanied with a slight decrease in the Seebeck coefficient in samples obtained from these attrition-milled precursors. Moreover, the use of an attrition milling process leads to a very important reduction in processing time (around 75%), together with a slight power factor improvement of around 10%, when compared to the classically prepared samples.

## 1. Introduction

Nowadays, thermoelectric (TE) materials stand out for harvesting wasted heat for direct power generation, without the need of mobile parts. For this type of application, it is necessary to produce materials with high TE performances, which are determined through ZT (TE figure of merit), involving the Seebeck coefficient (S), electrical resistivity (ρ), thermal conductivity (κ), and absolute temperature (T) parameters in the well-known expression, ZT = TS^2^/ρκ [1]. This relationship clearly points out that these high performances should be achieved by increasing the Seebeck coefficient and/or working temperatures or decreasing thermal conductivity and/or electric resistivity. Moreover, it can be divided into the thermal (T, and κ) and the electric (S, and ρ) parameters, with the relationship being between these electrical parameters and the thermoelectric power factor, PF = S^2^/ρ.

Semiconducting and intermetallic materials as Bi_2_Te_3_ [2], usually formed by heavy or toxic metals, are used to build high performance TE modules, which can be utilised in practical applications. In spite of the high ZT values exhibited by these materials, they can only work at relatively low temperature, due to their easy oxidation under air at high temperatures. Consequently, the high temperature waste heat sources are unusable for this type of modules. The discovery of high thermoelectric properties in Na_x_CoO_2_ [3] has allowed envisaging high-temperature waste heat recovery, as these kinds of materials can work at high temperatures and present high thermal and chemical stability. Moreover, the typical precursors used in these ceramics are cheaper, environmentally friendly, and more abundant in the earth’s crust than the ones used in classical thermoelectric materials [4]. These characteristics drastically gave a boost to the study of different oxide materials, searching for high thermoelectric properties. At present, several co-oxides are considered as good candidates for practical applications, such as Bi_2_Sr_2_Co_2_O_x_ or Ca_3_Co_4_O_9+δ_ [5,6]. On the other hand, these materials are usually prepared through the classical ceramic route, involving several calcinations and milling processes. Besides, the appearance of inhomogeneities and incomplete reactions after sintering are typical trademarks of this process. These drawbacks should be prevented through different approaches, which at the same time, can lead to the final products with improved properties to be industrially produced in a more economical way.

Taking into account these characteristics and previously published works about the effect of precursors sizes on the thermoelectric properties of ceramic materials [7], the aim of this work is to produce high-performance Ca_3_Co_4_O_9+δ_ materials using an easily scalable and environmentally friendly process, in a short processing time. Moreover, samples produced through the classical ceramic method will also be prepared to be used as reference.

## 2. Materials and Methods

Ca_3_Co_4_O_9+δ_ precursors were prepared using CaCO_3_ (≥99%, Panreac, Castellar del Vallès, Spain) and Co_3_O_4_ (99.5%, Aldrich, Saint Louis, MO, USA) commercial powders in the appropriate proportions. They were mixed thoroughly and milled using water media for 3 h in two different devices: attrition or ball mill. Attrition milling has been performed at 300 rpm using 1 mm diameter zirconia balls, while ball milling has been done with 10 mm diameter agate balls at 300 rpm using; in both cases, the milling media: precursor powder weight relationship is 10:1. These mixtures were used to produce the Ca_3_Co_4_O_9+δ_ materials via attrition milling or through the classical ceramic route after sintering, respectively. After drying the different suspensions under infrared radiation, they were manually ground to obtain fine powders, which were subjected to different calcinations: a two-step process (750 and 800 °C for 12 h) in the classical ceramic route, previously reported as the optimal calcination conditions [8]. On the other hand, attrition-milled precursors were calcined in a single step at 850 °C, as previously reported [9], optimizing the calcination time to only 1 h due to their small grain sizes. All these calcined powders were then cold-uniaxially pressed in form of pellets (15 × 3x ~ 3 mm^3^) under 400 MPa and sintered at 900 °C for 24 h in the classical solid-state route, and from 4 to 16 h at 900 °C for the attrition-milled precursors.

The precursors decomposition has been evaluated by DTA–TGA (TA Instruments, SDT Q600, New Castle, DE, USA) between room temperature and 800 °C at 10 °C/min heating rate. Phases identification has been made through XRD (Rigaku D/max-B X-ray powder diffractometer, Tokio, Japan) on the surface of sintered samples with CuKα radiation, and 2θ between 10 and 40 degrees, where the main diffraction peaks of Ca_3_Co_4_O_9+δ_ phase appear.

Microstructural observations were performed on precursor powders and longitudinal surfaces of sintered samples, using secondary electrons, in a field emission scanning electron microscope (FESEM, Zeiss Merlin, Jena, Germany) fitted with an energy dispersive spectrometry (EDS) device, (Oxford Instruments, Abingdon, UK). Apparent density has been determined on several samples for each preparation conditions, taking 4.677 g/cm^3^ as a theoretical one [10].

Electrical resistivity and Seebeck coefficient were simultaneously determined using the standard four-probe configuration, in the steady state mode, using an LSR-3 system (Linseis GmbH, Selb, Germany) between 50 and 800 °C under He atmosphere. With these data, PF has been calculated to determine the electrical TE performances.

## 3. Results and Discussion

### 3.1. Precursor Powders

**Figure 1:**
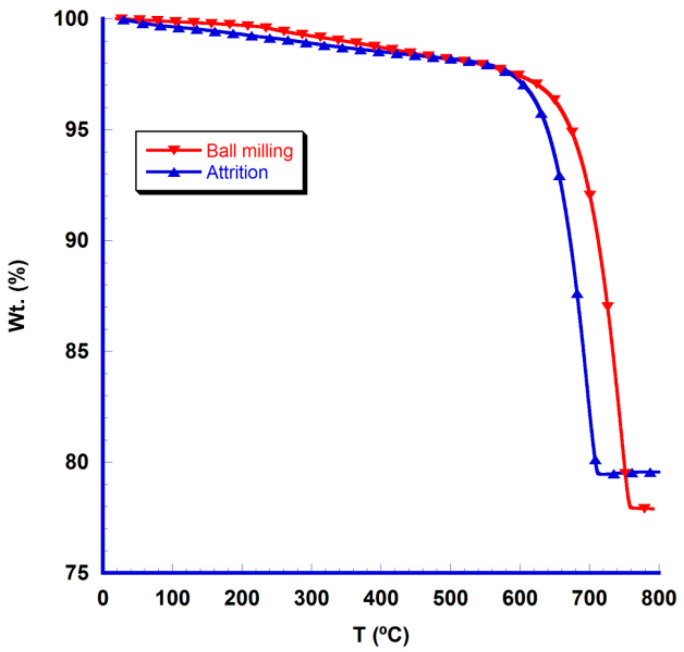
TGA graph of the dry precursors after milling.

TGA analysis performed on the different dry precursors provides information about their weight evolution with temperature, giving information about the decomposition and phase formation processes, as shown in Figure 1. As it can be easily observed in the graph, weight loss starts at a significant lower temperature for the attrition-milled precursors than for the ball-milled ones. Moreover, at high temperatures, ball-milled precursors lost more weight than the attrition ones. This effect can be explained taking into account the following reactions occurring during the thermal treatments:

CaCO_3_ + Co_3_O_4_ → CaO + Co_3_O_4_ + CO_2_ (1)

9 CaO + 2 Co_3_O_4_ + ½ O_2_ → 3 Ca_3_Co_2_O_6+δ_ (2)

3 Ca_3_Co_2_O_6+δ_ + 2 Co_3_O_4_ + ½ O_2_ → 3 Ca_3_Co_4_O_9+δ_ (3)

The first reaction is the main one in the calcination step, being responsible for the weight losses. On the other hand, the second reaction is happening, to a lower extent, during this calcination step, slightly increasing the samples weight. Consequently, it can be deduced that attrition-milled samples have higher reactivity than the ball-milled ones due to their smaller particle sizes, and the second reaction is produced in these precursors. Finally, the third reaction is mainly produced during the sintering step in both cases.

**Figure 2:**
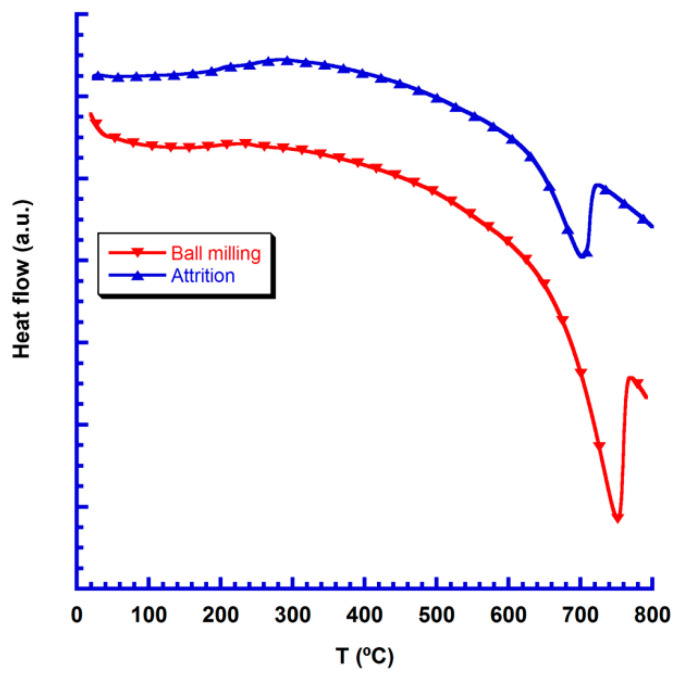
DTA graph of the dry precursors after milling.

DTA and TGA analyses have been simultaneously performed to evaluate the sign and magnitude of heat flow in the processes observed in the TGA graph. The sample evolution with temperature observed in the TGA analysis are confirmed through the DTA results displayed in Figure 2. In this plot, it can be easily observed that the endothermic peak appears at lower temperatures for the attrition-milled precursors. Consequently, the decomposition step of Ca carbonate is starting about 50–75 °C lower temperatures for the attrition-milled precursors, when compared to the ball-milled ones. Moreover, the heat flow is much lower in the attrition-milled precursors, pointing out to the lower CaCO_3_ decomposition energy, due to their much smaller particle sizes.

**Figure 3:**
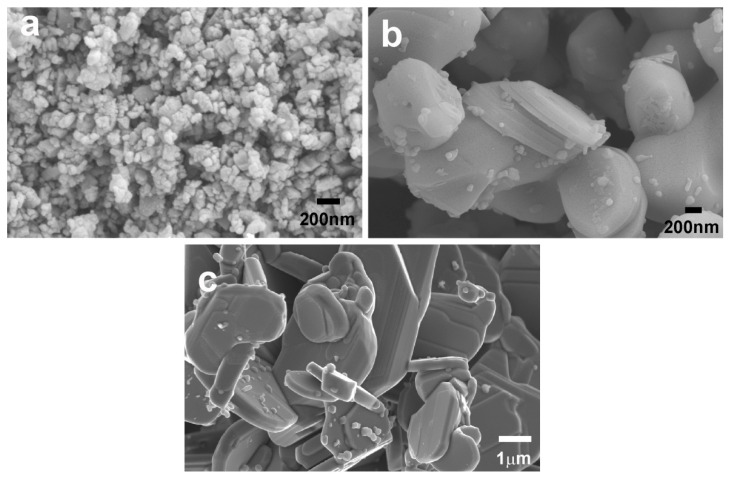
Representative FESEM micrographs obtained on (**a**) as-milled attrition powders; (**b**) attrition-milled powders after calcination at 850 °C for 1 h; (**c**) ball-milled powders after calcination at 750 °C for 12 h + 800 °C for 12 h.

These characteristics are illustrated in Figure 3, where the as-milled attrition powders, as well as the attrition and ball-milled ones after calcination, are shown. In the pictures, it can be easily observed that attrition-milled powders possess a mean size below 100 nm, being drastically increased after calcination at 850 °C for 1 h along the ab-plane, while their c-direction dimensions are still below 100 nm. These images clearly indicate that attrition-milled powders are very reactive, and that they preferentially grow along the ab-plane. On the other hand, ball-milled powders after calcination are much larger than the attrition-milled ones, along the ab-plane (>3 μm), and in the c-direction (>250 nm).

### 3.2. Sintered Materials

**Figure 4:**
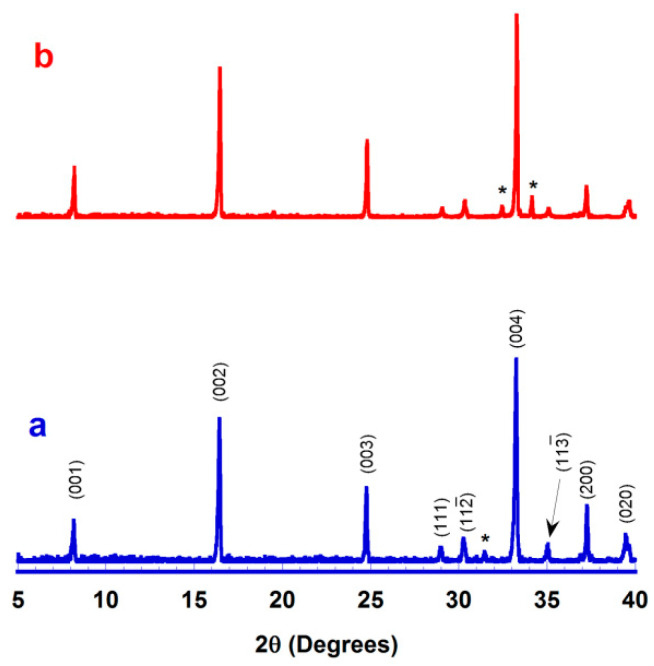
XRD patterns obtained on the surface of sintered materials produced by attrition milling and sintered for 4 h (**a**); ball milling (**b**). * indicates the reflections of Ca_3_Co_2_O_6+δ_ phase.

Representative XRD patterns performed on the surface of sintered samples are displayed in Figure 4. As it can be observed in the plot, most of the peaks are associated to the thermoelectric Ca_3_Co_4_O_9+δ_ phase, in agreement with previous studies [11]. Moreover, the most intense ones are assigned to the (00l) reflection planes, indicating a possible slight grain orientation, at least in the surface, due to the applied pressure during the ceramic conformation, as previously reported [12]. On the other hand, only minor peaks (indicated by *) belonging to the Ca_3_Co_2_O_6+δ_ phase [11] can be found, indicating that this phase is in very small proportion in all samples.

**Figure 5:**
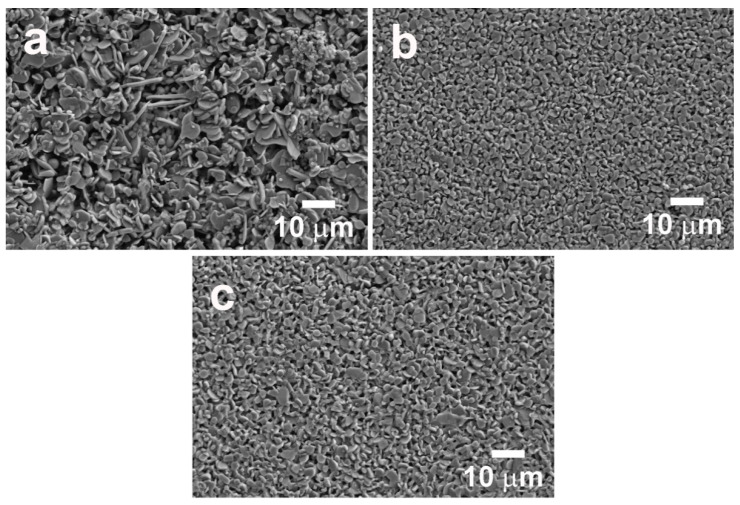
FESEM micrographs obtained on the surfaces of samples prepared from (**a**) ball-milled precursors; attrition-milled precursors sintered for (**b**) 4; (**c**) 16 h.

Representative FESEM images of sample surfaces are shown in Figure 5. As it can be seen in these micrographs, classically prepared samples show the coexistence of very large and small grains (Figure 5a). Moreover, they display much larger mean grain sizes than those prepared from attrition-milled precursors, independently of the sintering time (Figure 5b,c). This difference is due to the smaller precursors grain sizes processed through the attrition milling, as previously shown. Furthermore, samples prepared using attrition-milled precursors show very homogeneous grain sizes, and they are slightly larger when the sintering time is increased. Finally, these samples seem to have a lower porosity than the classically prepared ones, further decreasing when the sintering time is raised.

The microstructural features previously discussed are reflected in the sample’s density, determined through Archimedes’ method, which is 3.43 ± 0.02 g/cm^3^ for the samples prepared through the classical ceramic route, while it is between 3.50 ± 0.05 and 3.67 ± 0.03 g/cm^3^ for the ones produced from attrition-milled precursors, sintered for 4 and 16 h, respectively. Even with this enhancement of density values, they are still much lower than the produced through spark plasma sintering (4.35–4.58 g/cm^3^) [13,14], hot uniaxial pressing (4.13–4.52 g/cm^3^) [15,16], or two-step method (3.55–4.35 g/cm^3^) [17]. On the other hand, they are higher than the obtained in classically sintered samples (2.87–3.20 g/cm^3^) [18]. The observed behaviour in samples prepared in this work is clearly in agreement with the literature and the fact that the sintering temperature (900 °C) is low when compared to the one necessary to produce a liquid phase (1350 °C) [19], leading to very low densification during sintering process. In fact, when observing the phase equilibrium diagram, the limiting factor in the sintering conditions corresponds to the maximum Ca_3_Co_4_O_9+δ_ phase stability temperature (around 926 °C).

**Figure 6:**
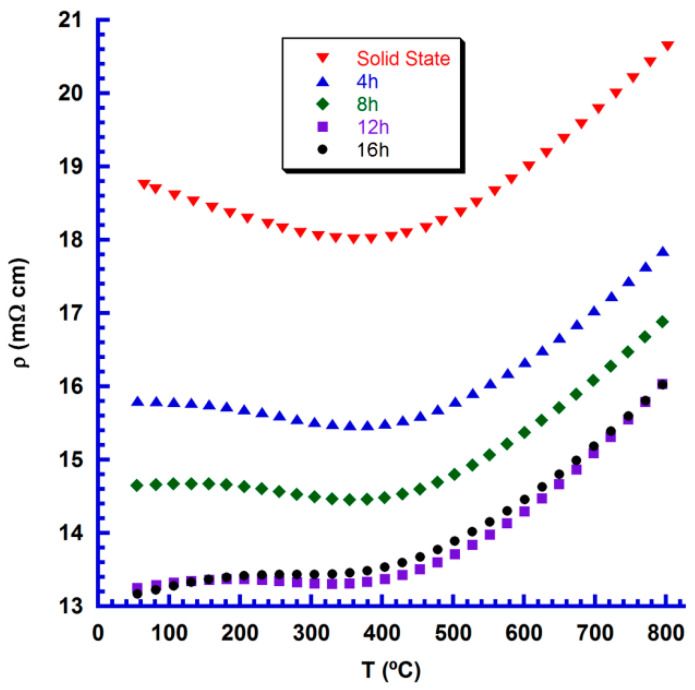
Temperature dependence of the electrical resistivity for the different Ca_3_Co_4_O_9+δ_ samples.

The evolution of electrical resistivity with temperature for all samples is shown in Figure 6. As it can be observed in the plot, all the samples show very similar evolution in the whole measured temperature range, in agreement with previously published results [20]. They show semiconducting behaviour from room temperature to around 400 °C, indicating hole hopping conduction from Co^4+^ to Co^3+^ [21] and metallic ones at higher temperatures. On the other hand, the use of attrition-milled precursors, with much smaller particle sizes and higher reactivity, probably lead to higher electrical conducting grain boundaries, explaining the lower electrical resistivity in these samples. Moreover, when increasing sintering time up to 12 h in these samples, electrical resistivity is further decreased, while it is maintained constant for longer sintering times. The minimum values determined at 800 °C (16 mΩ cm) in samples sintered for 12 h is about 25% lower than those measured in samples produced through the ceramic route in this work (20.6 mΩ cm) or previously published results (20–41.7 mΩ cm) [20,22,23]. Furthermore, they are similar to those reported in samples produced using solution methods (about 16 mΩ cm) [24] or by directional solidification (around 15 mΩ.cm) [25]. On the other hand, they are much higher than those obtained in textured materials produced by spark plasma sintering and measured along the ab-plane (6–8 mΩ.cm) [26,27,28]. It is worth mentioning that these textured materials show very high densities (>90% of the theoretical) and present very good grain alignment, explaining their very low electrical resistivity.

**Figure 7:**
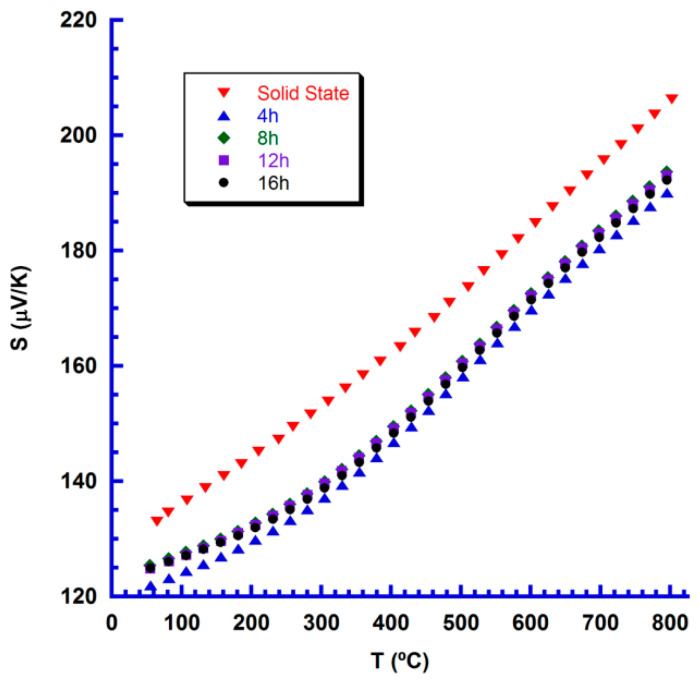
Temperature dependence of the Seebeck coefficient for the different Ca_3_Co_4_O_9+δ_ samples.

Figure 7 displays the variation of the Seebeck coefficient as a function of temperature for all samples. As it can be seen in the graph, S is positive in the whole temperature range for all samples, indicating a hole-dominating conduction mechanism. Moreover, samples produced through attrition milling show lower S values than the obtained in the ones prepared through the classical ceramic route, in agreement with the higher resistivity of these last samples. Furthermore, in spite of a slightly lower S values determined in samples sintered for 4 h, all the specimens produced from attrition-milled precursors display the same S values in the measured temperature range, which indicates that no changes in phase or oxygen content are produced after 8 h sintering. The highest value at 800 °C in these samples (~195 μV/K) is higher than the reported in spark plasma sintered samples (170–175 μV/K) [26], but slightly lower than the determined in laser floating zone grown materials (~205 μV/K) [25], which induces higher oxygen vacancies in the thermoelectric phase. Furthermore, they are in the order of those published for classically sintered materials (170–210 μV/K) [22,23].

**Figure 8:**
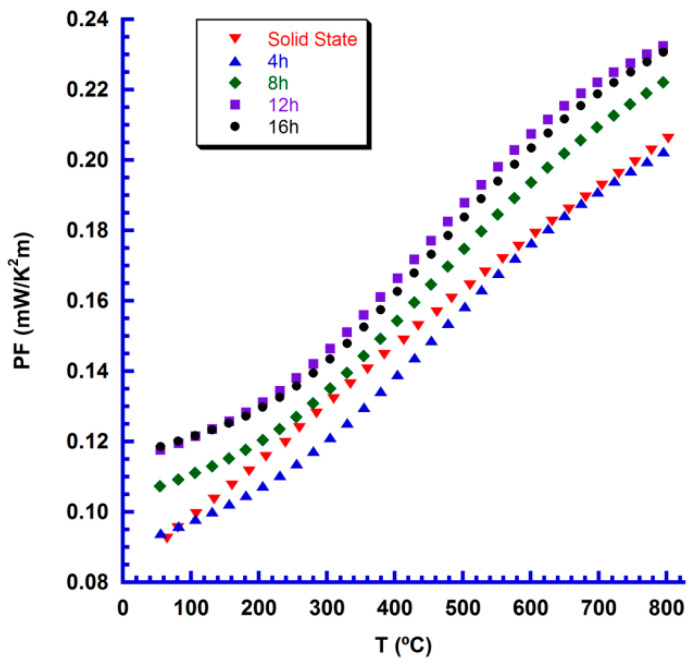
Temperature dependence of PF for the different Ca_3_Co_4_O_9+δ_ samples.

PF variation with temperature has been calculated for all samples, and the results are presented in Figure 8. From the data shown in this graph, it is clear that samples that sintered for 4 h show the same performances than those obtained in the classically produced ones. Moreover, when increasing sintering duration, their performances are raised up to 12 h and maintained constant for larger sintering times. The highest PF values at 800 °C (~0.23 mW/K^2^m), obtained in samples sintered for 12 h, are about 10% higher than the obtained through the classical ceramic route in this work, or in the literature (0.10–0.20 mW/K^2^m) [22,23]. On the other hand, they are much lower than the reported for textured materials produced by spark plasma texturing [26], or laser floating zone technique (~0.40 mW/K^2^m) [25]. In spite of these large differences, the samples produced in this work have been obtained through a very simple, economic, scalable, and environmentally friendly process, which may warrant their application in high-temperature thermoelectric modules.

## 4. Conclusions

In this work, a simple, economic, easily scalable, and environmentally friendly process to produce the Ca_3_Co_4_O_9+δ_ thermoelectric material has been presented. The use of attrition milling procedure has led to very reactive precursors due to their very small particle sizes, when compared to those obtained in the classical ceramic route. Smaller grain sizes and larger densities have been obtained with the attrition-milled precursors after sintering. Electrical resistivity has been drastically reduced in these attrition-milled samples, while Seebeck values have been only slightly decreased. Consequently, the highest power factor has been increased in around 10% at 800 °C for attrition-milled samples, when compared to the classical solid state sintered ones. Finally, the use of attrition-milled precursors has allowed a drastic reduction in processing time, together with a slight increase in thermoelectric performances.
